# Use of the triglyceride-glucose index (TyG) in cardiovascular disease patients

**DOI:** 10.1186/s12933-019-0982-2

**Published:** 2020-01-15

**Authors:** Javad Alizargar, Chyi-Huey Bai, Nan-Chen Hsieh, Shu-Fang Vivienne Wu

**Affiliations:** 1 Research Center for Healthcare Industry Innovation, National Taipei University of Nursing and Health Sciences, Taipei, 112 Taiwan; 2 School of Public Health, College of Public Health, Taipei Medical University, 250 Wu-Hsing Street, Taipei, 11031 Taiwan; 3 Department of Information Management, National Taipei University of Nursing and Health Sciences, Taipei, 112 Taiwan; 4 College of Nursing, School of Nursing, National Taipei University of Nursing and Health Sciences, Taipei, 112 Taiwan

**Keywords:** Cardiovascular diseases, Coronary artery disease, Risk factors, Secondary care, Triglyceride-glucose index

## Abstract

Da Silva et al. showed that the triglyceride-glucose (TyG) index was positively associated with a higher prevalence of symptomatic coronary artery disease (CAD). TyG has been used in healthy individuals as a marker of insulin resistance. The use of this index as a marker of atherosclerosis in cardiovascular disease (CVD) patients might be influenced by diabetes and the hyperlipidemic state that led to CVD. Certain considerations might be necessary before we conclude that the TyG index can be used as a marker of atherosclerosis in CVD patients. These factors can highlight the role of fasting blood glucose and triglyceride levels that are used in the TyG formula. Comparing the fasting blood glucose and/or triglyceride levels with the TyG index in these patients to show how much value the TyG index can add to clinical practice seems to be necessary. Conclusions of such studies might be biased by these facts. Stratification by CAD disease category cannot help achieve an understanding of the role of TyG in CVD. Correlations do not imply causation, so the use of the TyG index as an index in CAD patients is questionable.

We read with great interest the article by da Silva et al. [1] on how the triglyceride-glucose (TyG) index was associated positively with a higher prevalence of symptomatic coronary artery disease (CAD) and with the metabolic and behavioral risk factors that this study evaluated; the researchers concluded that this biomarker can be used as a marker for atherosclerosis.

Recent studies have widely used the TyG index as a marker of insulin resistance. It has been shown that a higher TyG index is associated with an increased risk of major adverse cardiac and cerebrovascular events in ST-elevation myocardial infarction (STEMI) patients undergoing percutaneous coronary intervention (PCI) [2] and that the risk of ischemic stroke correlates with a proportional and linear increase in the TyG index [3]. Zhao et al. [4] showed that an elevated TyG index is significantly associated with a higher risk of arterial stiffness and nephric microvascular damage. The TyG index is also used as a valuable biomarker for diabetes development, as it has shown an association with the risk of incident diabetes [5].

As da Silva et al. [1] mentioned, the TyG index is calculated as Ln (fasting triglycerides (mg/dl) × fasting blood glucose (mg/dl)/2). Although Moon et al. [6] stated that this index was proposed by Guerrero-Romero et al. [7] in 2010, we found that this index had been used by Simental-Mendia et al. [8] in 2008, using the same calculation for the first time in a large population-based cross-sectional study of healthy individuals. The rationale for why they used this index was that insulin resistance is a common cause of the increase in triglyceride and glucose levels in healthy individuals. In patients with apparent cardiovascular disease and diabetes, the use of this measure might be biased and have less diagnostic value than expected.

Da Silva et al. [1] included patients who had at least one cardiovascular disease (CVD) in the last 10 years and stratified them into three groups: (a) asymptomatic, (b) symptomatic and (c) treated for CAD. As they calculated the TyG index in all these patients, they observed a statistically significant difference only in the symptomatic group (Group b), as the higher TyG index tercile had a higher prevalence of symptomatic patients. They confirmed their conclusion by performing regression analyses on all groups and observing that these results were robust even after controlling for sex, age, and use of hypoglycemic, antihypertensive, anticoagulant and lipid-lowering agents.

It is worth noting that all of the patients included in their study were at risk of CAD because they had a previous history of CVD. Diabetes has been considered a main risk factor for CAD [9]. Triglycerides are well-known independent risk factors for CVD [10]. Da Silva et al. [1] did not report any statistics stratified by CVD, so there is a high possibility that many of the patients in the symptomatic group had the same characteristics regarding the controlled factors (included in the regression model, especially the use of hypoglycemic, antihypertensive, anticoagulant and lipid-lowering agents), so controlling for these variables does not greatly influence the conclusion.

The fact that more symptomatic patients belonged to the higher tercile of the TyG index can be easily explained by the fact that they had uncontrolled diabetes and/or hyperlipidemia, leading to high TyG index levels, as TyG has a direct relationship with triglycerides and glucose (based on the TyG formula). We can observe that this pattern is not seen in the other two groups: Groups a and c (asymptomatic and treated groups), as they have probably controlled these factors (good treatment and lifestyle habits in asymptomatic and good treatment regime and medications in the treated group).

Another point that is missing in this article is the fact that the authors could compare the diagnostic values of fasting glucose and triglyceride levels (and maybe the combination) with the TyG index and then try to show that the TyG index can have a better diagnostic value than fasting glucose and triglyceride levels. A medical doctor usually looks first at fasting glucose and triglyceride levels to screen high-risk patients, especially CVD patients. How can the TyG index add to the prognostic values of triglyceride and glucose levels? The fact that CVD is a dynamic and progressive disorder and that the initiation of treatment should be based on the specific situations of the patients makes using indexes such as the TyG index as prognostic markers less certain.

Using the TyG index in CVD patients can be easily biased by diabetes and hyperlipidemia, and these factors should be well controlled to justify its use as a biomarker. We should not infer reverse causality in the application of the TyG index in CVD patients.

## Data Availability

Not applicable.

## Abbreviations

TyG: triglyceride-glucose index
CVD: cardiovascular disease
CAD: coronary artery disease
STEMI: ST-elevation myocardial infarction
PCI: percutaneous coronary intervention
